# Foaming of PCL-Based Composites Using scCO_2_—Biocompatibility and Evaluation for Biomedical Applications

**DOI:** 10.3390/ma15113858

**Published:** 2022-05-28

**Authors:** Katarzyna Kosowska, Jan Krzysztoforski, Marek Henczka

**Affiliations:** Faculty of Chemical and Process Engineering, Warsaw University of Technology, Waryńskiego 1, 00-645 Warsaw, Poland; jan.krzysztoforski@pw.edu.pl (J.K.); marek.henczka@pw.edu.pl (M.H.)

**Keywords:** tissue engineering, supercritical carbon dioxide, foaming, poly(ε-caprolactone), porogen, scaffold

## Abstract

The process of foaming poly(caprolactone)-based composite materials using supercritical carbon dioxide was analyzed, especially in terms of the biocompatibility of the resultant materials. The influence of foaming process conditions and composite material properties on the functional properties of polymer solid foams, intended for artificial scaffolds for bone cell culture, was investigated. The relationship between wettability (contact angle) and water absorption rate as a result of the application of variable conditions for the production of porous structures was presented. For the evaluation of potential cytotoxicity, the MTT and PrestoBlue tests were carried out, and animal cells (mouse fibroblasts) were cultured on the materials for nine days. There was no toxic effect of composite materials made of poly(caprolactone) containing porogen particles: hydroxyapatite, crystalline nanocellulose, and graphene oxide on cells. The desired effect of the porogens used in the foaming process on the affinity of cells to the resultant material was demonstrated. The tested materials have been shown to be biocompatible and suitable for applications in biomedical engineering.

## 1. Introduction

Polymer foams represent an important branch of the science of polymer materials due to their wide range of useful properties and, hence, applications [1,2,3]. The literature presents the technology of foaming materials using supercritical fluids as a promising tool for the production of functional solid foams for cell culture. One of the methods for controlling the morphology of the resulting solid foams in the foaming process is the use of a particulate pore-forming substance (porogen), which has a significant impact on the effectiveness of nucleation [4]. In the literature on the subject, there are many reports on the use of polymeric materials enriched with uniformly dispersed nanoparticles of substances in the foaming process, constituting the sites of heterogeneous nucleation initiation [5,6,7,8,9,10,11,12,13,14,15]. These works conclude that the efficiency of nucleation strongly depends on the size and shape of the particles used, their surface properties, and the quality of particle dispersion. In 2018, very promising research results on the properties of microporous composite polylactide (PLA) foams containing cellulose nanocrystals were published [16]. The undoubted advantage of the resulting foams is a significant reduction in density compared to pure PLA foam, and greater interest in specialized areas of application, where the weight of the construct is important. In another study, a significant effect of the addition of graphene flakes to polycarbonate material on the functional properties of solid foams was demonstrated [17]. The dependence of the morphological properties of foams on the parameters of the two-step foaming process, as well as the amount and shape of the blowing agent used, was investigated, and the effectiveness of nucleation at the tested conditions was demonstrated. Liu et al. investigated the synergistic effect of foaming technology with the use of supercritical fluids and the specificity of nanocomposites [18]. This paper presents the evaluation of an effective method for producing a microporous polymer foam from polyvinyl alcohol enriched with graphene. In the foaming process, graphene sheets acted as sites of heterogeneous nucleation, facilitating the process of nucleation of bubbles.

The essence of the problem under consideration is the assessment of relationship between the conditions of the foaming process with the use of supercritical fluids, such as temperature, pressure, and saturation time, as well as the relationship between the polymer–gas or polymer composite–gas interface and the properties of the produced solid foams [19,20]. On the basis of a critical review of the literature and the results of independent experimental research, a significant role of the commonly used biodegradable polymer, namely poly(ε-caprolactone) (PCL), in the field of tissue engineering was demonstrated [11,15,20,21]. In addition, a promising method of foaming materials was identified, using carbon dioxide as a blowing agent for the efficient production of three-dimensional porous structures from poly(ε-caprolactone), characterized by high heterogeneity and relatively low mechanical strength. In many publications, the authors indicate the positive effect of pore-forming substances on the improvement of the properties of porous structures, mainly on the morphology and mechanical properties.

Recently, special attention has been paid to the characteristics of model solid foam with high utility in tissue engineering. The most important features were: appropriate solid foam morphology, including parameters such as porosity, pore shape and size, pore distribution, combination, and mechanical strength, including elasticity and mechanical strength, biodegradability, biocompatibility, wettability, non-cell toxicity, and many others [22,23,24,25,26,27,28,29,30,31,32,33,34,35,36,37,38,39,40,41].

The produced porous structures, used in biomedicine as a temporary support for developing cells, should be characterized by a number of requirements, both in terms of material and design, and should have biological features conditioning the adhesion, growth, and multiplication of cells, and supporting the expansion of the native extracellular matrix [23]. The role of the developed functional creations strictly depends on the location of their application. Therefore, it is important to ensure appropriate morphological and mechanical properties of the scaffold similar to those of the parent tissue by selecting appropriate materials and production methods [24]. The model scaffold should enable the differentiation of stem cells into cells that make up the regenerated tissue and ensure a stable and functional connection with the native [25]. Additionally, the created functional porous structures should enable the transport of nutrients and metabolites and stimulate vascular development until the functional tissue is completely rebuilt at the site of the defect [24]. In Table 1, the range of parameter variability characterizing the model solid foam applicable in biomedical engineering is presented.

By using a combination of many manufacturing techniques, as well as by using a composite material with a variety of characteristics, the porous structure characteristics can be determined [20].

The main goal of this study is to investigate the process of foaming materials using supercritical carbon dioxide, including to gain knowledge on the mechanisms of the foaming process using scCO_2_, to identify the influence of the foaming process parameters: saturation pressure, saturation temperature, and saturation time on porous structures properties.

The research presented here is the second part of the work on new functional composite materials with high utility in biomedical engineering. In the first part, the physicochemical and mechanical properties of the obtained porogen-enriched solid foams [4] were characterized, while in this part attention is paid to biological properties, namely biocompatibility and wettability. Moreover, it is important to investigate the possibility of a new application of various types of pore-forming substances, such as hydroxyapatite, cellulose, carboxymethylcellulose, and graphene oxide, and their mixtures as a raw material in the production of useful solid foams, and to prove the beneficial effect of their use on the course of the poly(ε-caprolactone) foaming process with the use of supercritical carbon dioxide and the effective modification of properties and improvement of the quality of the produced solid foams, increasing the usability of the produced porous materials in specialized practical applications. The specific purpose of this paper is the assessment of the usability of solid poly(ε-caprolactone) foams produced with the use of pore-forming substances, namely hydroxyapatite (nHA), cellulose (nC), carboxymethylcellulose (CMC), and graphene oxide (nGO), for tissue engineering applications, as well as the identification of process parameters with a key impact on the performance of these materials.

## 2. Materials and Methods

### 2.1. Materials

Experimental studies of the process of foaming the polymer material using supercritical carbon dioxide were carried out using composite materials based on poly(ε-caprolactone) (Sigma Aldrich, Poznań, Poland), a semi-crystalline, biodegradable polymer with a relatively low melting point. In the main part of the research, composite materials made of poly(ε-caprolactone) with addition of solid particles of porogens, i.e., hydroxyapatite (Sigma Aldrich, Poznań, Poland), nanocellulose (CelluloseLab, Fredericton, NB, Canada), and graphene oxide (Nanomaterials, Warsaw, Poland), were used. In order to obtain PCL composite materials, an appropriate amount of polymer granules was melted using a heating plate and appropriate amounts of pore-forming particles were added in batches, while ensuring continuous manual mixing of the resulting mixture. The share of individual components was determined experimentally, as a result of which the following shares were obtained: hydroxyapatite and nanocellulose 1–12% (*w*/*w*), graphene oxide 0.2–1.5% (*w*/*w*). Detailed information on the materials used can be found in the first part of this study [4]. The blowing agent for the composite material in the high-pressure tank was carbon dioxide (Linde Gaz, Poland) with a purity of 4.5 (99.995 vol.%). For biological studies, mouse fibroblasts from the L929 line (Sigma Aldrich, Poznań, Poland) and adherent cells obtained from the subcutaneous adipose tissue of C3H mice were used.

### 2.2. Methods

The process of foaming the materials was carried out in a specially designed and built high-pressure system. A detailed description and diagram of the system is presented in previous papers [19,20], especially the first part of this study [4]. The foaming process itself was carried out in a three-step batch process. In the first step, the material was placed in a high-pressure stainless-steel chamber, where it was melted and saturated with carbon dioxide under appropriate conditions of pressure, temperature, and saturation time. In this step, carbon dioxide penetrated the molten phase of the polymer matrix by diffusion. Then, in the second step, the mixture was cooled and in the third one the system was depressurized, accompanied by nucleation and growth of gas bubbles inside the polymer matrix. A more detailed description of the method of producing polymer porous structures is given in the first part of this work [4].

Composite polymer foams were made using the batch method of foaming materials using supercritical carbon dioxide. The foaming process itself occurs in three steps: saturation (step I)—P_sat_ = 9–18 MPa, T_sat_ = 50–100 °C, t_sat_ = 0.5–4 h, foaming (step II)—P_sat_ = 9–18 MPa, T_sat_ = 50–100 °C, t_sat_ = 0.08–0.5 h, system expansion (step III)—D_slow_ = 60 MPa/h, D_fast_ → ∞ MPa/h (instantaneous decompression to ambient pressure).

The obtained porous structures were tested with the use of specialized methods to assess biocompatibility and biological usefulness. Among the methods, the wettability test was used to determine the contact angle and the water absorption rate, the cytotoxicity tests MTT and PrestoBlue, and the cultures on the materials imaged on days 1, 3, 7, and 9 using scanning electron microscopy.

The porous structures most useful in tissue engineering should be characterized by appropriate wettability, which has a significant impact on the adhesion and orientation of the cells inhabiting them. As part of the research work, a test was carried out to determine the contact angle and liquid absorption rate of the obtained porous structures using the Contact Angle System OCA25 goniometer (Spectro-Lab, Warsaw, Poland). The research procedure included the preparation of the composite material (fragments of the sample with a thickness of ca. 3 mm and the flattest surface were cut from the cross-section), placing the sample on the goniometer table, forming a drop of distilled water with a volume equal to 5 μm, placing the drop on the composite material, and recording the film when no further penetration of drops into the composite was observed. The films were analyzed with the use of special software, determining the contact angles, droplet volume above the marked baseline and the test time from the moment the drop was deposited on the material to the end of liquid absorption. The absorptive capacity of the produced solid foams was assessed using the parameter called the rate of liquid absorption by the tested material, expressed by the following relationship:

(1) $$\frac{dV}{dt}=\frac{V_{i}-V_{f}}{t_{f}}$$

where *V_i_* denotes the initial drop volume (µL), *V_f_* the final drop volume (µL), and *t_f_* the total absorption time (min).

Cytotoxicity tests were performed in accordance with the procedure described in PNEN ISO 10993–5: 2009 (Biological evaluation of medical devices—Part 5: in vitro cytotoxicity tests) using two tests: MTT and PrestoBlue. The MTT and PrestoBlue assay is a colorimetric assay for assessing the metabolic activity of cells. The MTT reagent, 3-[4,5-dimethylthiazol-2yl]-2,5-diphenyl-tetrazolium bromide (tetrazolium yellow), is reduced to purple formazan in living cells. The PrestoBlue reagent is a ready-to-use resazurin-based solution. After entering living cells, resazurin is reduced to resorufin, a red colored and highly fluorescent compound. The control viability was 100% with a small value of the standard deviation of the sample. According to the standard, the material does not show toxic properties if the cell viability obtained as a result of the MTT or PrestoBlue test is at least 70%.

The last experimental method for assessment of biocompatibility was the cultivation of cells on the investigated porous materials. The culture procedure was started with the appropriate preparation of samples of the tested porous structures. The cells cultivated on the prepared materials are L929 cells, which belong to the group of adherent cells and grow in the form of a monolayer covering the growth surface in the culture vessel. It seemed necessary to prepare the materials in such a way that the cells maximally occupy the surface of the prepared fragments of solid foams, and that they do not develop on the remaining free surfaces of the culture vessel. From the tested materials, cuboids with dimensions of about 10 × 10 × 2 mm were cut so that when placed in a 24-well plate, they strictly adhered to the bottom surface of the well. Two samples of material were prepared for one culture time point. Cell culture on the materials was stopped after 1, 3, 7, or 9 days. Properly prepared samples were imaged using the SEM scanning electron microscope by Phenom World PRO (ThermoFisher Scientific, Warsaw, Poland).

## 3. Results and Discussion

To evaluate the biological properties, a series of toxicity tests were performed for the cells of the obtained solid foams produced from pure PCL. The results are presented in Table 2.

Based on the cytotoxicity tests performed, there was no toxic effect of the tested materials on the cells.

**Figure 1:**
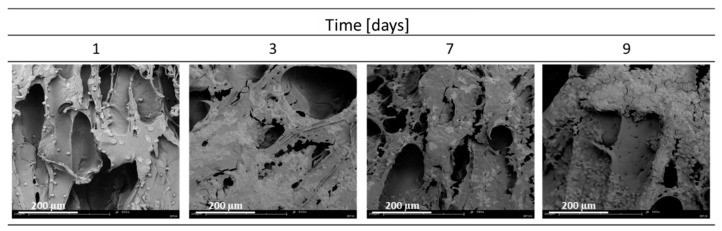
Cultured cells on a solid foam of pure poly(ε-caprolactone) produced at T_sat_ = 70 °C, P_sat_ = 18 MPa and t_sat_ = 1 h on days 1, 3, 7 and 9 of culture.

Cell cultures were then performed on solid foams produced under different conditions of temperature pressure and saturation time. SEM images of the cell culture performed on the materials produced at T_sat_ = 70 °C, P_sat_ = 18 MPa, t_sat_ = 1 h on days 1, 3, 7, and 9 of culture are shown below (Figure 1).

The medium seems to be not quite suitable for the seeded cells, as they are rounded and refuse to flatten out. They grow in clusters, which is where they have managed to create a niche for themselves. Perhaps they lack a factor that would encourage them to expand to the entire surface of the material, so it is worthwhile to produce a composite material that shows greater bioactivity.

Next, the results are presented separately for all the porogens applied, in the range of the concentrations used, for the following production conditions: variable saturation pressure ∆P for constant temperature T_sat_ = 70 °C, saturation time t_sat_ = 1 h and system expansion D_fast_, variable temperature ∆T for constant saturation pressure P_sat_ = 18 MPa, saturation time t_sat_ = 1 h and system expansion D_fast_, variable time of saturation ∆t for constant pressure P_sat_ = 18 MPa, temperature T_sat_ = 70 °C and system expansion D_fast_. The investigated set of parameters was determined experimentally on the basis of the research presented in [4].

### 3.1. Nanohydroxyapatite

**Figure 2:**
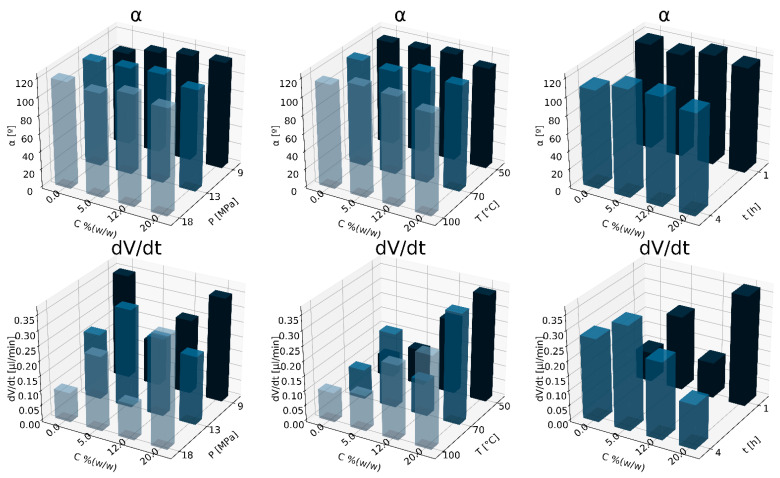
Influence of nHA concentration for variable conditions of the foaming process: pressure P_sat_ (9–18 MPa), T_sat_ (50–100 °C) and saturation time t_sat_ (0.5–4 h) on the contact angle (α) and absorption rate (dV/dt).

In Figure 2, the relationships of wetting angle and absorption rate at varying conditions of hydroxyapatite concentration, pressure, temperature, and saturation time are shown. As the hydroxyapatite concentration increased, the contact angle decreased, and the absorption rate increased.

In Table 3, the results of cytotoxicity tests of solid foams made of PCL/nHA composite material produced at various conditions of pressure, temperature, and saturation time are summarized. Based on the collected data, no cytotoxic effect of the materials was observed.

**Figure 3:**
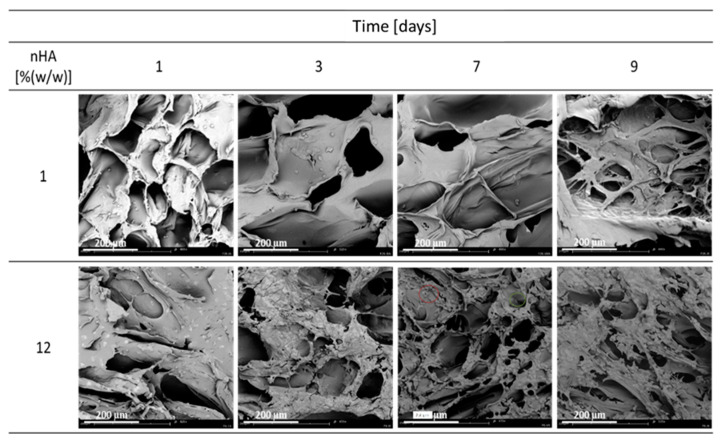
Cell culture on composite materials containing 1 and 12% (*w*/*w*) nHA produced at the conditions of T_sat_ = 70 °C, P_sat_ = 18 MPa, t_sat_ = 1 h on 1, 3, 7 and 9 days.

In Figure 3, SEM photos from the conducted cell culture on the tested materials are presented. For 1% (*w*/*w*) nHA, the cells are nicely flattened after 24 h. After 72 h, the culture appears to be dying out as the cells are more rounded than flattened. However, after nine days, they are scattered throughout the material, but they do not form clusters or flatten out. The differences may result from the variety of samples from the tested material. Moreover, it seems that the cells grow better than on pure PCL material. Material containing 12% (*w*/*w*) nHA seems to be a better environment for cell growth and development than pure PCL, as cells flatten out and form clusters, i.e., divide and occupy almost the entire available surface.

### 3.2. Nanocellulose

**Figure 4:**
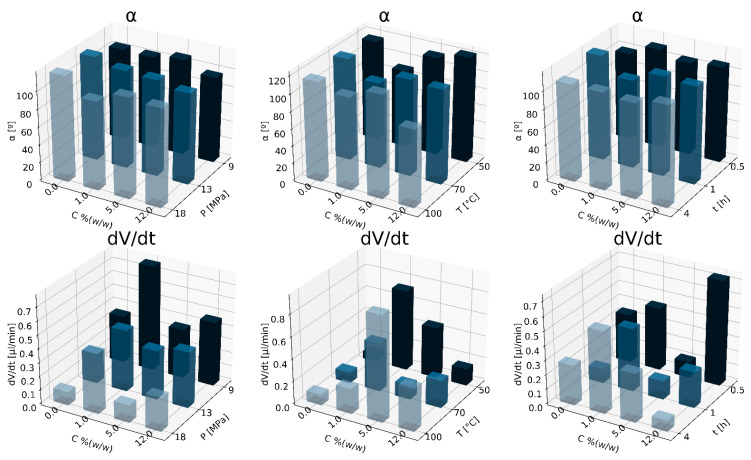
Influence of nC concentration for variable conditions of the foaming process: pressure P_sat_ (9–18 MPa), T_sat_ (50–100 °C) and saturation time t_sat_ (0.5–4 h) on the contact angle (α), absorption rate (dV/dt).

In Figure 4, the effect of nC concentration on parameters describing the wettability of PCL/nC composite solid foams is shown. A decrease in the saturation pressure and an increase in the nC concentration led to an increase in the wettability of solid foams. By using the addition of a porogen, the rate of water absorption by the produced solid foam, and thus the wettability, increases. A decrease in the saturation pressure and an increase in the nanocellulose concentration led to an increase in the wettability of solid foams.

In Table 4, the results of cytotoxicity tests of solid foams made from PCL/nC-based composite materials produced at various conditions of pressure, temperature, and saturation time are summarized. No toxic effects of the tested materials on cells were identified.

**Figure 5:**
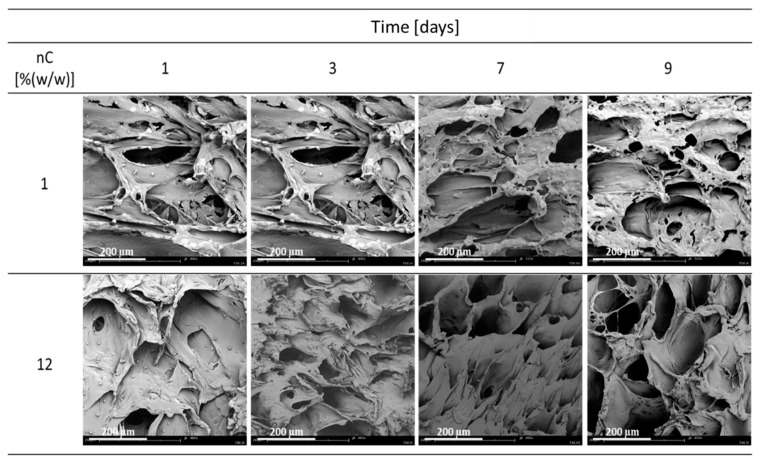
Cultivation of cells on composite materials containing 1 and 12% (*w*/*w*) nC produced at the conditions of T_sat_ = 70 °C, P_sat_ = 18 MPa, t_sat_ = 1 h on days 1, 3, 7 and 9.

In Figure 5, SEM pictures of cell culture on materials containing nC are shown. At 1% (*w*/*w*) nC, cultures look good, cells flatten and divide. On the other hand, the number of cells is slightly smaller than when 12% nHA is used. Moreover, using higher concentrations of nC, namely 12% (*w*/*w*), an unfavorable effect on cells is observed as they appear to be poorly flattened. On the first and third day, cells were observed that began to flatten or to die, and in the following days only single cells are visible.

### 3.3. Nanographene Oxide

**Figure 6:**
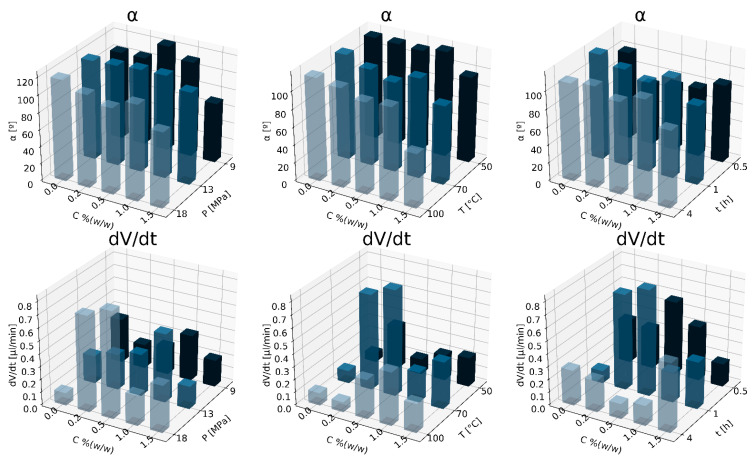
Influence of nGO concentration for variable conditions of the foaming process: pressure P_sat_ (9–18 MPa), T_sat_ (50–100 °C) and saturation time t_sat_ (0.5–4 h) on the contact angle (α), absorption rate (dV/dt).

In Figure 6, the results of the effect of nGO concentration on the wettability parameters, i.e., the contact angle and absorption rate, for different manufacturing conditions of solid foams are shown. In general, the use of nGO leads to an increase in the wettability of the resulting composite solid foam. The addition of nGO increases the wettability of the porous structure at a variable value of the saturation temperature, while the highest values of the absorption rate in the tested range of operating variables were obtained as a result of saturation of the composite at 70 °C. The use of nGO results in structures with higher wettability.

In Table 5, the results of cytotoxicity tests of solid foams produced from PCL/nGO-based composites are summarized. The results of the cytotoxicity test confirm the possibility of using nanographene oxide as a pore-forming substance in the production of porous structures for biomedical applications. On the basis of the presented data, no toxic effects of the composite material used on the cells were identified.

**Figure 7:**
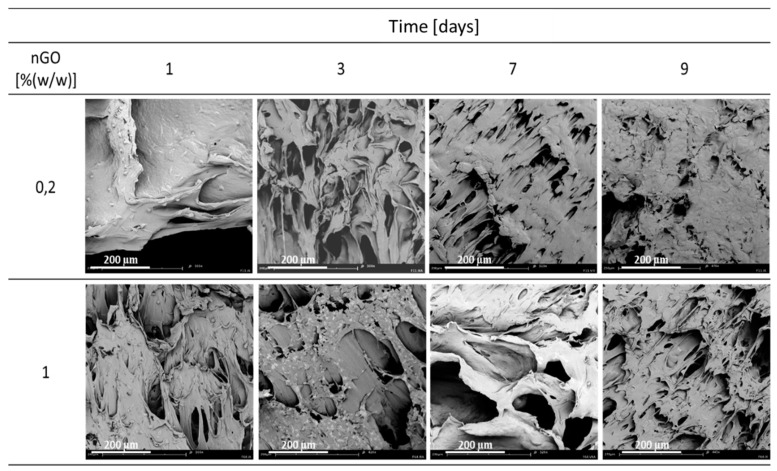
Cell culture on composite materials containing 0.2 and 1% (*w*/*w*) nGO produced at the conditions of T_sat_ = 70 °C, P_sat_ = 18 MPa, t_sat_ = 1 h on 1, 3, 7 and 9 days.

In Figure 7, SEM images of cell cultures on PCL/nGO based composite materials produced at the conditions of T_sat_ = 70 °C, P_sat_ = 18 MPa, t_sat_ = 1 h on days 1, 3, 7, and 9 are shown. On the tested material containing 0.2% (*w*/*w*) nGO, cells developed well and flattened out after 24 h. Moreover, after nine days, the material seems to be completely covered with cells. For 1% (*w*/*w*) nGO after 24 h, cells behave similarly to those cultured on material containing 0.2% (*w*/*w*) nGO. Based on the image after three days, one can expect that in the following days the growth on the composite material with 1% (*w*/*w*) nGO should be much better than in the case of the material with a lower concentration of graphene oxide.

### 3.4. Discussion

The process of foaming materials using supercritical carbon dioxide is one of the methods for producing functional solid foams that can be used in biomedical engineering as artificial scaffolds for cell culture. There are many reports in the literature on the use of composite materials based on polymers enriched with various types of auxiliaries for the production of highly useful porous structures. The use of these substances often guarantees the possibility of using solid foams in specific applications [5,6,7,8,9,10,11,12,13,14,15,16,17,18]. A particularly important part of this work is the research on biological properties and the possibility of using structures in biomedical engineering [24,29,33,40,41,44]. In a previous paper [4], the influence of the conditions of the foaming process and the characteristics of the materials used, including the degree of fragmentation, on the functional properties of porous structures was investigated. Parameters describing the morphology were determined: pore diameter, pore volume density or porosity, and mechanical properties: Young’s modulus and maximum compressive strength, which are within the acceptable range for the use of the resulting solid foams as scaffolds in regenerative medicine.

In the previous paper [4], solid foams produced by the method of foaming polymeric materials with using supercritical carbon dioxide were characterized in terms of morphology and mechanical properties. The effectiveness of the production process and the possibility of using the obtained porous structures in biomedical engineering as scaffolds for bone cell culture were assessed.

In this paper, the non-cytotoxic nature of the materials was demonstrated and the influence of the manufacturing conditions on the wettability of the resulting structures was examined. The rate of water absorption by the porous structure increased with the increase in the concentration of the porogen. In addition, the most intense changes in wettability were identified for changes in the saturation pressure compared to other foaming conditions. The results of the culture experiment showed an increase in the affinity of cells for the material for the material containing blowing agents compared to the pure polymer solid foams. The most promising culture results were shown for the use of graphene oxide in both low and high concentrations. The tested materials have been shown to be biocompatible and suitable for applications in biomedical engineering. In practice, many other biological tests are used to prove that the material is cell-friendly. Therefore, additional biological tests, such as live-dead assay or testing the stability and biodegradability of the material, are planned as part of further work.

## 4. Conclusions

The biocompatibility of PCL composites with nHA, nC, and nGO foamed with scCO_2_ was studied and compared with the biocompatibility of pure PCL foams foamed under analogous conditions. Parameters describing wettability of solid foams, i.e., contact angle and absorption rate, strongly depend on both the conditions of manufacturing process and concentration of blowing agent additives (nHA, nC, and nGO) used. No toxic effects of the substrate on the cells were observed, neither for solid foams produced from pure PCL nor the polymer-based composite material. By culturing on foams made of pure PCL, the necessity of creating a medium supplemented with a suitable factor to increase its bioactivity was demonstrated. The material containing nHA seems to be a better environment for cell growth and development than pure PCL. The addition of a portion of nC stimulates cell growth, whereas an increase in porogen concentration leads to the inhibition of this growth and cell death. The addition of nGO also stimulates cells to grow. Additionally, the use of even a high concentration of nGO does not inhibit this growth. Based on the evaluation of morphological and mechanical properties, as well as bioactivity parameters, it was shown that the material that best meets the requirements for biomedical applications is the composite enriched with nHA (low concentrations—1–5% (*w*/*w*) nHA) and nGO (tested concentration range—0.2–1.5% (*w*/*w*) nGO). The tested materials have been shown to be biocompatible and suitable for applications in biomedical engineering.

## Figures and Tables

**Table 1:** Required properties of solid foams for biomedical applications.

Property	Parameter	The Range of Variability	Application	References
Morphology	Porosity	80%	Biomedical engineering	[28,29,30,31,32,42]
	Pore size	5 μm	Newly formed blood vessels	[36]
		5–15 μm	Connective tissue cells in the growth phase	
		20 μm	Hepatocytes	
		20–125 μm	The skin of an adult human	
		100–350 μm	Bone tissue	
		>500 μm	Fibrous vascular tissue	
		Micropores	Tissue engineering	[37]
		100 μm	Bone tissue	[28]
		450 μm	Bone tissue	[43]
		≤100 μm	Connective tissue	
		Micropores (<50 μm) and macropores (150–300 μm)	Fibrous cartilage tissue	
		<100 μm	Bone tissue	[26]
		100–500 μm	Blood vessels	
		200–350 μm	Bone tissue	[30]
		100–350 μm	Bone tissue	[36]
Mechanical	Young’s modulus	7–30 GPa	A creature packed with bones	[25,29,39]
		0.02–0.8 GPa	Bone spongy creature	
	Mechanical strength	100–230 MPa	A creature packed with bones	
		2–12 MPa	Bone spongy creature	
Biological	Biocompatibility	Optimal biotolerance, non-toxicity, no genetic mutations in the surrounding cells and no inflammation	Biomedical engineering	[24,29,33,40,41,44]
	Biodegradability	The rate of degradation of the implanted material was adjusted to the rate of regeneration of damaged tissue	Biomedical engineering	[24,29,33]

**Table 2:** Effect of PCL foaming process execution conditions: pressure, temperature, saturation time, decompression rate on cell viability.

		MTT	Presto Blue
		CELL VIABILITY [%]	
	CONTROL	100 (±0.10)	100 (±0.02)
P [MPa]	9	94.6 (±0.11)	95.2 (±0.15)
	18	97.5 (±0.05)	97.7 (±0.05)
t [h]	0.5	93.2 (±0.01)	91.3 (±0.02)
	6	98.0 (±0.03)	87.3 (±0.25)
T [°C]	50	84.0 (±0.06)	91.0 (±0.10)
	100	87.0 (±0.04)	95.0 (±0.01)
D	D_slow_	91.4 (±0.01)	84.2 (±0.12)
	D_fast_	87.8 (±0.02)	87.6 (±0.02)

**Table 3:** Influence of the concentration of the applied additive nHA and the foaming process conditions: pressure P_sat_, temperature T_sat_, saturation time t_sat_ on the viability of cells.

		C [%(*w*/*w*)]	MTT	Presto Blue
			CELL VIABILITY [%]	
	CONTROL		100 (±0.10)	100 (±0.02)
P [MPa]	9	1	80 (±0.11)	78.0 (±0.25)
		5	78 (±0.05)	85.3 (±0.04)
		12	82 (±0.01)	80.0 (±0.03)
	18	1	89 (±0.03)	90.5 (±0.01)
		5	86.5 (±0.06)	82.3 (±0.03)
		12	84.5 (±0.04)	85.6 (±0.11)
t [h]	1	1	87.5 (±0.01)	80.8 (±0.01)
		5	92.4 (±0.02)	98.4 (±0.02)
		12	95.0 (±0.10)	88.2 (±0.11)
	4	1	98.0 (±0.11)	85.3 (±0.02)
		5	93.0 (±0.05)	87.6 (±0.06)
		12	88.0 (±0.01)	82.6 (±0.02)
T [°C]	50	1	75.0 (±0.03)	80.5 (±0.10)
		5	85.0 (±0.06)	82.6 (±0.05)
		12	82.5 (±0.04)	80.6 (±0.02)
	100	1	93.5 (±0.01)	92.5 (±0.11)
		5	87.0 (±0.02)	90.5 (±0.05)
		12	85.5 (±0.10)	86.2 (±0.02)

**Table 4:** Influence of the concentration of the applied additive nC and the foaming process conditions: pressure P_sat_, temperature T_sat_, saturation time t_sat_ on the viability of cells.

		C [%(*w*/*w*)]	MTT	Presto Blue
			CELL VIABILITY [%]	
		CONTROL	100 (±0.10)	100 (±0.02)
P [MPa]	9	1	98.7 (±0.11)	88.2 (±0.04)
		5	92.3 (±0.05)	80.4 (±0.05)
		12	95.0 (±0.01)	80.6 (±0.10)
	18	1	88.4 (±0.03)	85.4 (±0.11)
		5	87.5 (±0.06)	85.4 (±0.05)
		12	85.5 (±0.04)	90.4 (±0.10)
t [h]	1	1	89.4 (±0.01)	92.4 (±0.04)
		5	84.5 (±0.02)	90.4 (±0.10)
		12	86.4 (±0.10)	88.2 (±0.02)
	4	1	96.4 (±0.11)	92.4 (±0.01)
		5	96.7 (±0.05)	90.4 (±0.06)
		12	88.5 (±0.01)	80.4 (±0.05)
T [°C]	50	1	78.4 (±0.03)	82.6 (±0.11)
		5	77.6 (±0.06)	80.4 (±0.03)
		12	82.5 (±0.04)	81.3 (±0.10)
	100	1	86.4 (±0.01)	82.4 (±0.03)
		5	88.7 (±0.02)	81.5 (±0.06)
		12	74.55 (±0.10)	82.5 (±0.01)

**Table 5:** Influence of the concentration of the applied additive nGO and the foaming process conditions: pressure P_sat_, temperature T_sat_, saturation time t_sat_ on the viability of cells.

		C [%(*w*/*w*)]	MTT	Presto Blue
			CELL VIABILITY [%]	
		CONTROL	100 (±0.02)	100 (±0.10)
P [MPa]	9	0.2	95.0 (±0.11)	88.2 (±0.02)
		0.5	92.0 (±0.05)	90.4 (±0.01)
		1	88.0 (±0.01)	90.4 (±0.05)
		1.5	87.0 (±0.03)	85.4 (±0.03)
	18	0.2	96.4 (±0.06)	86.4 (±0.10)
		0.5	86.7 (±0.04)	84.5 (±0.06
		1	89.2 (±0.01)	90.4 (±0.10)
		1.5	76.4 (±0.02)	92.1 (±0.06)
t [h]	1	0.2	88.7 (±0.10)	90.5 (±0.02)
		0.5	89.6 (±0.11)	87.5 (±0.01)
		1	82.6 (±0.05)	86.5 (±0.03)
		1.5	74.2 (±0.01)	80.4 (±0.02)
	4	0.2	82.6 (±0.03)	86.2 (±0.01)
		0.5	86.2 (±0.06)	80.2 (±0.05)
		1	78.5 (±0.04)	78.4 (±0.03)
		1.5	98.5 (±0.01)	82.5 (±0.02)
T [°C]	50	0.2	92.6 (±0.02)	80.2 (±0.01)
		0.5	88.4 (±0.10)	89.1 (±0.06)
		1	82.6 (±0.01)	90.2 (±0.01)
		1.5	86.4 (±0.03)	80.5 (±0.02)
	100	0.2	88.6 (±0.06)	86.4 (±0.05)
		0.5	89.6 (±0.04)	82.6 (±0.01)
		1	87.5 (±0.01)	84.5 (±0.06)
		1.5	84.6 (±0.02)	87.3 (±0.01)

## Data Availability

Not applicable.
